# The Connection between Chronic Liver Damage and Sporadic Alzheimer’s Disease: Evidence and Insights from a Rat Model

**DOI:** 10.3390/brainsci13101391

**Published:** 2023-09-29

**Authors:** Ruchi Jakhmola Mani, Nitu Dogra, Deepshikha Pande Katare

**Affiliations:** Proteomics and Translational Research Lab, Centre for Medical Biotechnology, Amity Institute of Biotechnology, Amity University, Noida 201301, India; rjakhmola@amity.edu (R.J.M.); twinkledogra@gmail.com (N.D.)

**Keywords:** Alzheimer’s disease, liver, brain, ALT, APP, junk food

## Abstract

Junk foods are typically low in essential nutrients, such as vitamins, minerals, and antioxidants. They are also loaded with trans fats and saturated fats, which can increase the level of triglycerides in the blood. High triglyceride levels can contribute to the development of non-alcoholic fatty liver disease (NAFLD), a condition where excess fat accumulates in the liver. A high intake of junk foods can lead to insulin resistance, a condition where the body’s cells become less responsive to insulin. A diet lacking in nutrients and loaded with unwanted toxins can impair the liver’s ability to detoxify harmful substances and damage its overall function. It is known that the regular consumption of junk food can be linked to memory impairment and cognitive decline. Several studies have shown that diets high in unhealthy fats, sugars, and processed foods can negatively impact brain health, including memory function. In this study, Wistar rats were used to model Late-Onset Alzheimer’s Disease (LOAD), which was inspired by knowledge of the liver–brain axis’s role in causing dementia. The model mimicked junk-food-induced liver–brain damage, and was developed by using the toxins d-galactosamine, ethanol and d-galactose. To begin with, the model rats demonstrated insulin resistance, a characteristic of LOAD patients. Glucose levels in both the brain and liver tissues were significantly elevated in the model, paralleling clinical findings in LOAD patients. High glucose levels in the brain lead to the increased production of advanced glycation end-products (AGEs), which, along with amyloid beta, harm neighbouring neurons. Histopathological analysis revealed deformed glial nodules, apoptotic neurons, and amyloid plaques in the brain section in the later stages of the disease. Simultaneously, the liver section displayed features of cirrhosis, including an effaced lobular architecture and the extravasation of red blood cells. Liver enzymes ALT, AST and ALP were consistently elevated with disease progression. Furthermore, immunohistochemistry confirmed the presence of amyloid precursor protein (APP) in the diseased brain. The positive expression of Hypoxia-Inducible Factor 3-Alpha (HIF3A) in the brain indicated hypoxic conditions, which is consistent with other LOAD studies. This model also exhibited damaged intestinal villi and excessive bowel and urinary incontinence, indicating malnutrition and a disturbed gut microbiome, which is also consistent with LOAD patients. Bioinformatics analysis on serum protein suggests a few affected molecular pathways, like the amyloid secretase pathway, androgen/oestrogen/progesterone biosynthesis, the apoptosis signalling pathway, the insulin/IGF pathway-protein kinase B signalling cascade, the Metabotropic glutamate receptor group I pathway, the Wnt signalling pathway, etc. Behavioural analysis confirmed memory decline and the loss of muscle strength with disease progression. Overall, this rat model of LOAD sheds valuable light on LOAD pathology and highlights the potential link between liver dysfunction, particularly induced by the excessive consumption of junk food, and LOAD. This study contributes to a deeper understanding of the complex molecular mechanisms involved in LOAD, paving the way for new possibilities in therapeutic interventions.

## 1. Introduction

AD is often called a disease of the elderly and mostly affects the older age group (65 years and above). Alzheimer’s Disease International (ADI) has estimated that currently, worldwide, over 50 million people are suffering from dementia, and this is expected to reach 152 million by 2050 [1]. In India alone, it is projected that around 7.6 million people will be suffering from AD by 2030 [2]. AD pathophysiology initiates in the hippocampal region of the brain, which is involved with memory and learning, and later spreads to the other parts of the brain. Many factors have been deliberated over pertaining to the onset of AD, but its actual cause is still unknown. A few studies have verified the role of other metabolic diseases like NAFLD (non-alcoholic fatty liver disease), cirrhosis, Diabetes Mellitus, Obesity, etc., in contributing to and progressing the symptoms of AD [3,4].

The relationship between liver health and cognitive decline in later life is now widely acknowledged [5]. If liver health continuously deteriorates without any pathogenic or external factors, it is likely attributed to the consumption of unhealthy food and an unhealthy lifestyle. The inclusion of junk food in our daily diet leads to the depletion of vitamins and minerals, an abnormal hormonal profile, and DNA oxidation [6]. Junk food is a colloquial term used to describe food and beverages that are typically high in calories and low in nutritional value. These foods are often processed, highly palatable, and often contain excessive amounts of unhealthy ingredients, such as saturated and trans fats, processed sugars, and alcohol. These dietary habits ultimately result in insulin resistance, which, in turn, triggers metabolic dysfunction. Sweetened junk foods contain significant amounts of sucrose and fructose, often in the form of high-fructose corn syrup (HFCS), an artificial sweetener [7]. Remarkably, even a 10% sucrose solution has been found to impair hippocampal memory [7]. In a study involving rats fed a sucrose-rich diet, elevated levels of TNF alpha and IL-1 beta were observed, indicating inflammation in the hippocampus. Additionally, increased NRF1 levels suggest oxidative stress in the hippocampus [8]. Recent advancements in molecular and computational biology have demonstrated that Alzheimer’s Disease is a complex condition that cannot be addressed by focusing solely on a single gene or pathway. AD involves a combination of processes, including decreased acetylcholinesterase levels, reduced insulin sensitivity, the accumulation of advanced glycation end-products (AGEs), the dysfunction of APOE, etc. Hence, it is of utmost importance to develop an animal model of Late-Onset Alzheimer’s Disease (LOAD) that accurately replicates the pathology observed in humans.

We have developed and published an in silico hypothesis to understand the role of the liver–brain axis in LOAD progression [9]. In the present work, a novel LOAD model was created in Wistar rats to validate the liver–brain-axis theory, which proposes that the liver is behind the massive neurodegeneration in AD.

## 2. Methodology

### 2.1. Development of Late-Onset AD (LOAD) or Sporadic AD

#### Wistar-Rat Model

Adult male Wistar rats of 250–300 gm weight were chosen for this study. The 21-week-old Wistar rats were grouped into six rats per cage and acclimatized to laboratory conditions under standard conditions (21 ± 2 °C; 55 ± 5 % relative humidity; and 12 h of light/dark cycles). The animals were given free access to a regular diet and water ad libitum. Permission to perform all animal experiments under the guidelines of the Institutional Animal Ethics Committee (503/CPCSEA) was obtained. The study group was as follows:

Group 1: Control (n = 10).

Stage 1 (Duration: 30 days), n = 10

Stage 2 (Duration 60 days), n = 10

The subjects were monitored daily for their behavioural changes and were sacrificed every month. Brain and liver samples were stored for histopathological analysis, and biochemical and proteomics evaluations. Dosing was conducted in 3 steps. Step 1: Oral dose of D-Galactosamine (10 mg/kg) body weight, then 2 days gap. Step 2: Oral dose of ethanol (8%/355 mL/60 kg body weight), then 3 days gap. Step 3: d-Galactose (100 mg/kg body weight). Then, 2 days gap, and step 1 through step 3 was repeated for 60 days.

### 2.2. Behavioural Tests

#### 2.2.1. Morris Water Maze Test

The Morris water maze test was conducted for investigating the memory and cognition of the subjects. Subjects were given prior training on the platform in one specific quadrant of the tank. After disease progression, the test was repeated, and data were collected pertaining to time taken to reach the platform and time spent in the specific quadrant.

#### 2.2.2. Rotarod Test

This test was conducted for examination of muscle strength and body balance. Subjects were given prior training on the moving rod for approximately 120–150 s. The rats were placed on a moving rod with an accelerating speed (10–40 rpm). After disease progression, rats were evaluated again for 150 s. Their frequency of falling from the rod and their falling time was recorded.

#### 2.2.3. Elevated Y-Maze Test

The elevated Y–Maze Test is an important behavioural test for determining the compliance of rats to explore a new area. In this test, the hippocampal, septum, forebrain and cortex regions of the brain are majorly involved. Rodents usually prefer to explore a new arm of the maze relative to returning to the one that had formerly been visited. In the Y-shaped-maze test, three opaque plastic arms are placed at an angle of 120° from each other. The subject should show a tendency to enter a less-recently visited arm. The percentage of alteration was calculated by recording the number of arm entries and triads.

#### 2.2.4. Hole-Board Test

The hole-board apparatus comprised of a wooden box measuring 60 cm × 60 cm. The height of the walls was 30 cm, and it was raised 28 cm above the ground. Nine holes were (4 cm) were made in the apparatus. The floorboard of the box was marked and divided into four areas along with one central area, which was masked with black tape. The apparatus was situated in a testing room with diminished white light. At the start of the test, the subject was positioned in one corner, facing the centre. Each test lasted for 10 min and at the end, the subject was placed into the carrying box. Between each trial, the floor was cleaned with 70% alcohol solution.

## 3. Sample Preparation

Wistar rats were sacrificed using cervical dislocation under the effect of anaesthesia in an ethical way. The subjects were dissected and their brains, livers, intestines, and kidneys were washed in cold saline (0.1 M, pH 7.4), and the tissue was stored at −80 °C. Some parts of the tissue were stored in formalin for histopathological analysis and some in 0.1 M PBS of pH 7.4 for biochemical analysis. Serum samples of the control and the diseased groups were also stored for further use.

### 3.1. Biochemical Assessments

#### 3.1.1. Determination of Acetylcholinesterase (AChE) Activity

The AChE activity was estimated by using the protocol of Ellman et al. [10]. For AChE-activity measurement, the reaction volume of 1.56 mL included 1.3 mL sodium phosphate buffer, 0.05 mL DTNB, 10 µL ATC and 0.2 mL supernatant. The absorbance was measured at 412 nm. The AChE activity was expressed as micromoles of ATC hydrolysed/min/mg protein.

#### 3.1.2. Determination of Glucose Levels

Glucose estimation was performed using the Glucose Oxidase–Peroxidase (GOD-POD) method in an endpoint mode by using an Autospan Glucose Kit [11]. The absorbance was measured at 505 nm. Glucose levels were expressed as mg/dL.

#### 3.1.3. Determination of Catalase Activity (CAT)

The CAT activity was analysed using the Claiborne method [12]. A total of 50 mM phosphate buffer (pH 7.4), 19 mM hydrogen peroxide (H_2_O_2_), and 50 µL PMS (post-mitochondrial supernatant) were part of the reaction, in a total volume of 3.0 mL. Changes in absorbance were measured at 240 nm. The CAT activity was expressed as nmol H_2_O_2_ consumed/min/mg protein.

#### 3.1.4. Determination of Lipid Peroxidation (LPO)

Lipid peroxidation assay was performed using a modified Ohkawa method [13]. The total mixture was prepared using 0.15 mL of 10% tissue homogenate, 0.70 mL of 0.67% thiobarbituric acid in PBS, 18 µL of 10 mM butylated hydroxytoluene (BHT) in ethanol and 2.130 mL of 1% orthophosphoric acid. The absorbance of the reaction mixture was recorded at 535 nm. The malondialdehyde (MDA) levels were expressed as nmoles of MDA formed/mg protein/min.

#### 3.1.5. Determination of Total Glutathione (GSH)

The GSH assay was performed using following the method of Jollow et al. (1974). The total reaction volume consisted of 0.1 mL of supernatant, 2.7 mL of 0.1 M phosphate buffer (pH 7.4) and 0.2 mL of DTNB. The coloured end product was measured at 412 nm [14]. The activity was expressed as micrograms/mg protein.

#### 3.1.6. Aspartate Aminotransferase (AST) and Alanine Aminotransferase (ALT)

The activity of AST and ALT were determined using kits and the modified IFCC method of Schumann et al. The absorbance was measured kinetically at 340 nm [15]. The AST and ALT levels were expressed as UI/L.

#### 3.1.7. ALP Activity Was Determined Using the Kits and Method of Tietz [16]

The change in absorbance was recorded kinetically at 405 nm. The ALP levels were expressed as UI/L.

### 3.2. Histopathological Analysis

Wistar rats were sacrificed, and their brains, livers and intestines were quickly removed and preserved in buffered 10% formalin for histopathological studies. The tissues were embedded in paraffin wax, and later, blocks were sectioned into 4 µm thick slides. The sections were stained with Haematoxylin (H) and Eosin (E). The photomicrographs of slides were examined under an Olympus CKX41SF inverted microscope system (Olympus Corporation, Tokyo, Japan).

### 3.3. Fiji Analysis

FiJi software 2.9.0 was used to analyse histopathological images. It calculates the average size of a cell, area fraction and total area. It is also useful in calculating the cell count in an image [17].

### 3.4. LC/MS QTOF

Liquid chromatography was performed on a ACQUITY UPLC system (Waters Corporation, Milford, Massachusetts, USA. The separation of all samples was performed on ACQUITY UPLC BEH C18 column (Waters, UK) (150 mm × 2.1 mm × 1.7 μm). A gradient elution program was run for the chromatographic separation, with mobile phase A (0.1% formic acid in water), and mobile phase B (0.1% formic acid in acetonitrile) as follows.


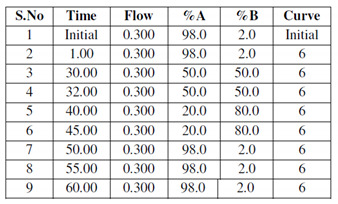


A SYNAPT G2 QTOF (Waters, UK) equipped with an electrospray ionization (ESI) source was used for mass spectrometric detection. The sample analysis was performed in a positive mode.

#### 3.4.1. Mass Spectrometric Raw-Data Analysis

The raw data acquired from the instrument were processed using PLGS software 3.0.2, within which data processing and a database search were performed. The sequence in FASTA format was used for searching the peptides present in the sample using the following search parameters in the software.

#### 3.4.2. Search Parameters

Peptide Tolerance (ppm): 50

Fragment Tolerance (ppm): 100

Missed Cleavages: 1

Modification: Carbamidomethyl_c, Oxidation_m

Database: Rat Protein (Swissprot)

Search engine: PLGS

#### 3.4.3. In-Solution Digestion Protocol

- 100 μg of extracted protein was taken for digestion.
- The sample was treated with 100 mM DTT at 95 °C for 1 hr, followed by 250 mM IDA at room temperature in the dark for 45 min.
- The sample was then digested with Trypsin and incubated overnight at 37 °C.
- The peptides were extracted in 0.1% formic acid and incubated at 37 °C for 45 min.
- The solution was centrifuged at 10,000× *g*, and the supernatant was collected into a separate tube.
- The resulting sample was vacuum-dried and dissolved in 20 μL of 0.1% formic acid in water; a 10 μL injection volume was used on C18 UPLC column for separation of peptides.
- The peptides separated on the column were directed to a Waters Synapt G2 Q-TOF instrument for MS and MSMS analysis.
- The raw data were processed using MassLynx 4.1 WATERS. The individual peptides’ SMS spectra were matched to the database sequence for protein identification on PLGS software v4.0 (Protein Lynx Global Server), WATERS.

### 3.5. Immunohistochemistry Analysis

The IHC analysis of amyloid-beta precursor protein, insulin receptors, B-cell lymphoma-2 and hypoxia-inducible factor 3 subunit alpha was performed using the following primary and secondary antibodies.

Primary antibodies: anti-beta amyloid (1:200 dilution); anti-insulin receptor alpha (1:250); and anti-Bcl-2 (1:200) and HIF-3 α (1:500).

Secondary antibody: Rabbit anti-Rat igG (HRP) conjugated (1:500).

#### 3.5.1. Preparation of Solutions and REAGENTS

1. Ethanol, anhydrous denatured, histological grade (100%, 95%, 85%, 75%);

2. Xylene;

3. Washing buffer;

4. Antigen Retrieval Solution;

5. Hydrogen Peroxide 3%;

6. Hematoxylin QS;

7. Permanent Mounting Medium.

#### 3.5.2. Deparaffinization and Rehydration

- The slides were heated in an oven for 5 min at 60 °C, followed by three times of washing in xylene for 10 min. Further slides were washed 3 times in 100% ethanol for 3 min. Then, slides were washed in 95% ethanol for 1 min. Again slides were washed, in 85% ethanol for 1 min, and finally, slides were washed in 75% ethanol for 1 min.
- The slides were rinsed in d.w. for 5 min. For the retrieval of Antigen, the slides were immersed in antigen retrieval solution and kept in a pressure cooker at 120 °C for 2.5 min. Then, the slides were cooled down at room temperature. Later, the slides were rinsed with distilled water for 2 min.

#### 3.5.3. Staining

- The tissue was covered with 3% hydrogen peroxide for 15 min to inactivate the activity of endogenous peroxidase. The slides were washed with PBST for 2 min. Later, primary antibodies were applied evenly to each section and incubated at room temperature for 90 min.
- Again, the slides were washed with PBST solution (3 times) for 2 min. Then, the secondary antibody was applied evenly and incubated at room temperature for min.
- The slides were washed with 1X PBST (2 min).
- Freshly prepared DAB substrate was added to the sections, and the tissue sections were incubated with the substrate at room temperature for the development of suitable staining (5 min).
- Each section was thoroughly washed with distilled water.
- The sections were counterstained with haematoxylin QS for 3 min, and again rinsed with distilled water.
- In the final stage, slides were washed in 75% ethanol (1 min), then with 85% ethanol (1 min), then with 95% ethanol (1 min), followed by washing in 100% ethanol (1 min). This was followed by 3 changes in xylene for 1 min.
- The coverslips were mounted on slides and were allowed to dry overnight at room temperature.
- The results of the slides were analysed with a microscope.

### 3.6. Protein–Protein Interaction-Network Building

The dysregulated proteins observed in both the liver and brain tissues were investigated further for the construction of protein–protein interaction (PPIs) networks. The STRING database is a repository of all known/validated PPIs and also predicts the interaction between the protein nodes in a network based on genomic patterns such as co-occurrence, gene neighbourhoods, domain fusion, text mining, etc. Functional enrichment of the network was also performed, and later, k-means clustering was performed to cluster the dysregulated genes according to the similarities of their biological pathways.

### 3.7. Statistical Analysis

The data are expressed as mean +/− standard deviation. All the analysis was performed using Origin software 2020 v 2022. The comparison of the means of the control and the treated Wistar rats was carried out using a one-way ANOVA followed by the Tukey test, with * *p* < 0.0001 as the limit of significance.

## 4. Results and Discussion

In the current study, the sporadic AD model was developed with the aim of exhibiting a condition caused due to excessive junk-food eating and excessive drinking habits; therefore, after a literature review, ethanol and d-galactose were chosen as promoters, along with DGalN. Ethanol and d-galactose have also been used by many other researchers to model AD. Ethanol is known for modelling liver injuries, and d-galactose is known for its use in aging models. According to recent human pathological reports, there are a few new symptoms which are important and need to be addressed in a sporadic AD model. The symptoms are bowel incontinence, urinary incontinence, a loss of muscle strength, excessive sugar in the brain tissue, the dysfunction of liver enzymes, etc., along with memory decline. We developed a novel rodent model for sporadic AD by using the combination of three compounds, i.e., d-Galactosamine, ethanol and d-Galactose.

### 4.1. Behavioural Analysis

#### 4.1.1. Morris Water Maze Test

**Figure 1:**
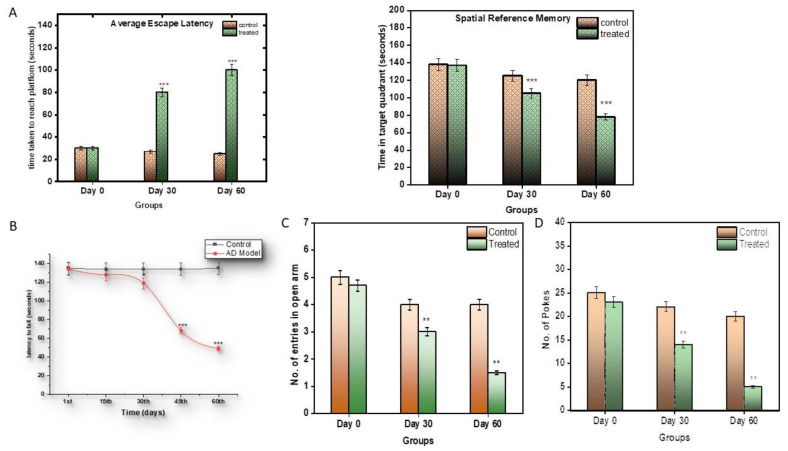
(**A**) Graphical representation of significant changes in the capacity of subjects to return to the platform, as a reference to its memory. (**B**) Graphical representation of significant changes in the capacity of subjects to hold the rod, as a reference to its muscular strength. (**C**) Graphical representation of Elevated Y-Maze Test; (**D**) graphical representation of hole-board test. Values are mean ± SD 10 rats in each group; *** *p* < 0.0001 compared to control.

The Morris water maze test was conducted to analyse the memory and cognition of the control and treated subjects. Prior to the model development, all subjects were given similar training to find and climb the platform (Figure 1A).

After the induction of the model, the control and the treated groups were compared. It was observed that the control subjects took around 30 s to explore and reach the platform, while, as the model progressed, it took almost 90 s for the treated subjects to find and climb the platform (Figure 1A), and they stayed for around 80 s in the quadrant where the platform was located, longer than the control subjects.

#### 4.1.2. Rotarod Test

The rotarod test was performed to assess the muscular strength of the control and the treated subjects. The control subjects were observed to hold the moving rod for around an average of 130 s, while the treated subjects were not able to hold the rod properly after the 1st month, as the disease progressed. The treated subjects, from the second month, were able to hold the moving rod only for an average of 50 s (*p* < 0.0001), less than the control subjects (Figure 1B).

#### 4.1.3. Elevated Y-Maze Test

In the Elevated Y-maze Test, the efficiency of the treated subjects decreased (Figure 1C). The willingness of the subjects to explore the new environment decreased with respect to the control subjects.

#### 4.1.4. Hole-Board Test

In the hole-board test, a similar behavioural pattern was observed. Anxiety and stress levels decreased in the treated groups (Figure 1D).

### 4.2. Biochemical Analysis

Biochemical estimations were conducted on both the serum and tissue samples of the control and the treated subjects.

#### 4.2.1. Acetylcholinesterase Assay

**Figure 2:**
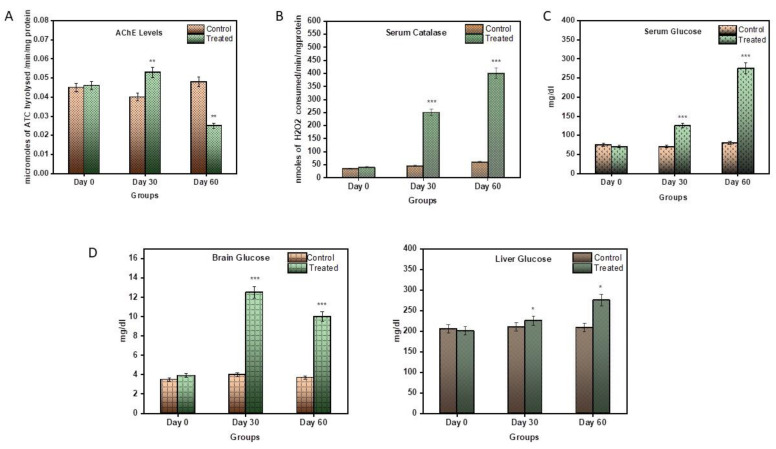
(**A**) Graphical representation of significant changes in AChE activity between the control and stages of disease progression in terms of the micromoles of ATC hydrolysed/min/mg protein. (**B**) Graphical representation of significant changes in the catalytic activity in serum samples between the control and stages of disease progression in terms of nmoles of H_2_O_2_ consumed/min/mg protein. (**C**) Graphical representation of significant changes in the glucose levels in serum samples between the control and stages of disease progression in terms of mg/dL. (**D**) Graphical representation of significant changes in the glucose levels in the brain and liver tissue between the control and stages of disease progression in terms of mg/dL. Values are mean ± SD 10 rats in each group; ** *p* < 0.0001 compared to control.

AChE levels were measured in the brain tissue of the control and the treated subjects. In the treated subjects, during the 1st month, the AChE levels increased to a significant amount of 0.0587 µmoles/min/mg (*p* < 0.0001 **), but their levels of AChE declined eventually by the 2nd month to 0.0259 µmoles/min/mg, and were significantly lower than those of the control subjects. Thus, during the progression of the disease, it was seen that the AChE levels first increased and then decreased compared to the control group (Figure 2A). A reduction in AChE levels is suggestive of reduced neuronal signalling.

#### 4.2.2. Catalase Activity

The catalase estimation was performed to assess the antioxidant capacity of the blood serum catalase enzymes. The serum of the treatment subjects displayed an increased spike in catalase activity compared to the control subjects (Figure 2B). During the first month, its activity rose to 247.97 nmole/min/mg, and then eventually to 412.22 nmole/min/mg in the 2nd month. An increase in catalase activity can be indicative of increased inflammation in the blood.

#### 4.2.3. Serum Glucose

Serum glucose levels were also measured in the control and the treated subjects. Glucose is an important parameter other than as a fuel: its presence can be useful in the assessment of glucose uptake by cells and, hence, an indication of insulin resistance. Neurons take energy from glucose to survive and perform. During the first month, there was a little spike in serum glucose levels, i.e., 109 mg/dL, but in the second month, the glucose levels rose to 255.67 mg/dL (*p* < 0.0001). This is a very significant rise in serum glucose (Figure 2C).

#### 4.2.4. Tissue Glucose

Brain and liver glucose levels were also measured in this model. Glucose is an important parameter in AD, as neurons take energy from glucose in order to survive and perform. Also, the liver is the storage site of glucose. Therefore, glucose levels are also an indicator of neuronal health, as well as liver health. In the brain, during the 1st month, the glucose levels increased significantly, i.e., 12.19 mg/dL, compared to the control, and later, by the 2nd month, came down to 11.78 mg/dL (Figure 2D), which was, again, very significantly higher than the control (*p* < 0.0001). Likewise, in the liver, the glucose levels increased very minutely in the 1st month, i.e., 211.09 mg/dL, and then increased gradually to 265.21 mg/dL in the second month, much higher than to those of the control subjects (Figure 2D).

#### 4.2.5. Serum ALT and AST

**Figure 3:**
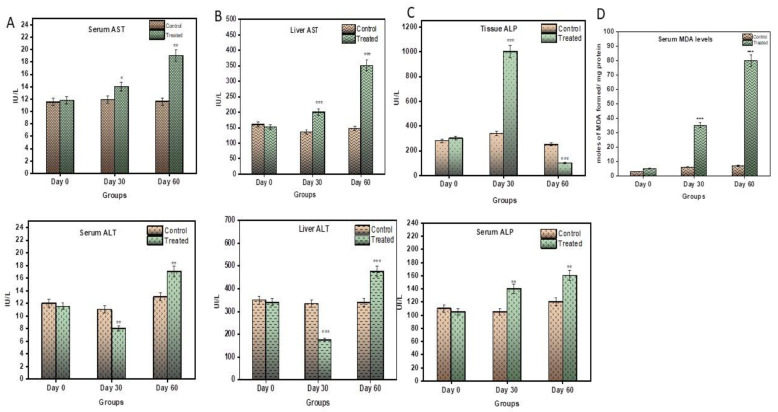
(**A**) Graphical representation of significant changes in (a) ALT and (b) AST levels between control and stages of disease progression in terms of UI/L. (**B**) Graphical representation of significant changes in (a) ALT (b) AST level between control and stages of disease progression in terms of UI/L. (**C**) Graphical representation of significant changes in ALP levels in (a) serum (b) liver tissue between control and stages of disease progression in terms of UI/L. (**D**) Graphical representation of significant changes in MDA levels between control and stages of disease progression in terms of nmoles of MDA formed/mg protein. Values are mean ± SD 10 rats in each group, * *p* < 0.05 and ** *p* < 0.0001 compared to control.

Serum Alanine transaminase (ALT) and Aspartate transaminase (AST) are major enzymes used in the assessment of liver health. ALT is a more direct assessment of liver health, while AST is an indicator of inflammation in hepatocytes. In the current model, ALT levels decreased in the 1st month to 7.2 UI/L and increased significantly to 16.32 UI/L by the end of the 2nd month (*p* < 0.0001) (Figure 3A). Likewise, the AST levels increased gradually as the disease progressed (*p* < 0.05).

#### 4.2.6. Tissue ALT and AST

ALT and AST assessments were also performed on liver tissue. The ALT levels were observed to decrease sharply in first month to 134.52 UI/L and then increase significantly to 179.02 UI/L (*p* < 0.0001) (Figure 3B). AST, on the contrary, was observed to significantly increase with disease progression (Figure 3B).

#### 4.2.7. Alkaline Phosphatase (ALP) Assay

Alkaline Phosphatase (ALP) is an enzyme mainly formed by liver cells. Its unusual presence in the blood indicates livery injury. The levels of serum ALP in the treatment group increased gradually and significantly, to 162.07 UI/L, compared to the control subjects (*p* < 0.0001), by the end of the 2nd month (Figure 3C). Contrary to this, the ALP in the liver tissue remarkably increased in the 1st month to 984.92 UI/L (*p* < 0.0001), and then there was a significant decrease in ALP to 110.86 UI/L by the end of the 2nd month.

#### 4.2.8. Lipid Peroxidation Assay (LPO)

Lipid peroxidation assay was conducted on serum samples to assess the level of inflammation in the body. The level of inflammation was directly proportional to the MDA formed in the sample. As seen in Figure 3D, the levels of MDA rose significantly and gradually in the treated subjects (*p* < 0.0001) in both the 1st and 2nd month.

#### 4.2.9. Histopathological and Fiji Analysis

**Figure 4:**
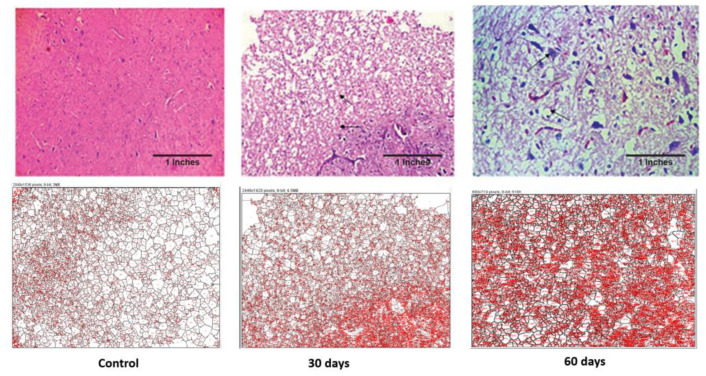
Photomicrographs of histopathological changes in the hypothalamus region of brain tissue of a Wistar rat. Figure displays control, 30th-day and 60th-day brain samples (40×).

Histopathological examination was performed for brain and liver samples collected on 30th day and 60th day of disease progression. As seen in Figure 4, the control brain has normal architecture. The treated subject, at the 30th day, shows white matter oedema and loss of astroglial cells. The brain section retrieved from the subject on the 60th day shows deformed glial nodules and apoptotic neurons. It also shows apoptotic neurons with occasional degenerated neurons and a few amyloid plaques. Severe vacuolization in this stage within the cell matrix is also an indication of cell death.

**Figure 5:**
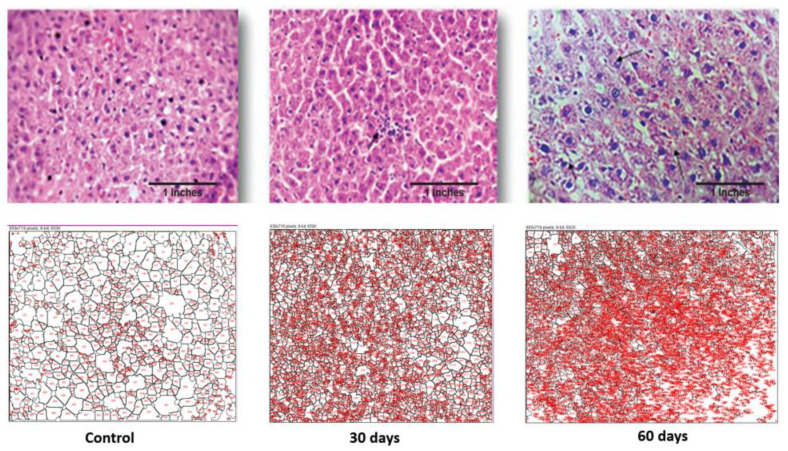
Photomicrographs of histopathological changes in the liver section of a Wistar rat. Figure displays control, 30th-day and 60th-day sample (40×).

The control liver showed a normal architecture, while the 30th-day sample showed occasional spotty necrosis (Figure 5). The sample retrieved from the 60th-day subject displayed a mildly effaced lobular architecture, an oval nucleus with coarse chromatin and prominent nucleoli and the extravasation of RBCs. The histopathology images were analysed using FiJi software 2.9.0, and the data are displayed in Table 1.

As seen in Table 1, the average sizes of both the brain and liver cells were reduced as the disease progressed. In the case of the brain, the total area was increased but the average cell size reduced, which is indicative of inter-cell vacuolization and is a contributing factor towards cell apoptosis. Additionally, in the case of the liver cells, both their total surface area and the average cell size reduced drastically as the disease progressed. This displays the increased liver cell apoptosis seen in an advance stage of liver toxicity.

#### 4.2.10. Bowel and Urinary Incontinence: A Novel Observation Mimicking Human AD Symptoms

**Figure 6:**
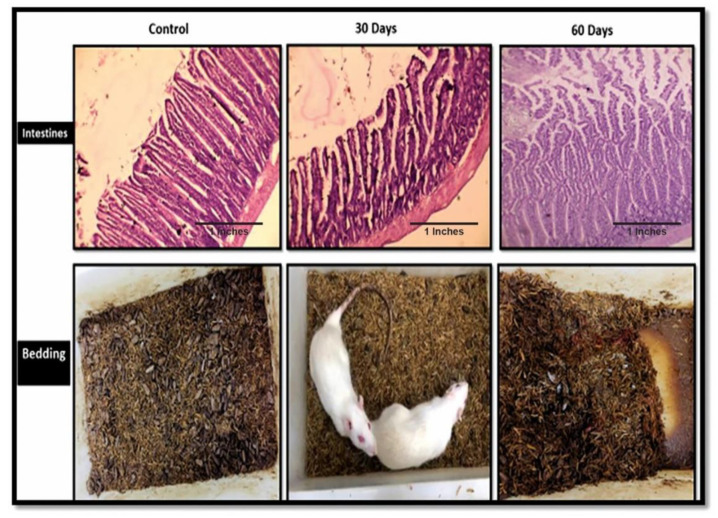
The histopathology images of the intestines of the control, 30th-day subjects and 60th-day subject, along with their cages showing their bowel movements.

The current sporadic AD model closely mimicked a few of the other human symptoms of AD patients. During the experiments, it was observed that the bowel movements of subjects were fine until the 30th day of the disease progression, but after the 30th day the subjects displayed increased urination and liquid bowels, and by the end of the second month, the subjects displayed excessive bowel incontinence. One subject also succumbed to death due to excessive urine and bowel movements. Figure 6 clearly shows the damage to the villi of the intestines with disease progression.

The model in totality mimicked the various human symptoms of AD, like increased levels of glucose and reduced levels of AChE in the brain in severe stages of the disease. The behaviour analysis revealed a decline in cognition, as well as weak muscular strength. The animals exhibited bowl inconsistency and high urination, which are the major symptoms of an advance stage of AD. Further, we performed differential protein expression analysis using Nano-LCMS to understand the molecular mechanism of the LOAD model.

#### 4.2.11. Differential Protein Expression Analysis for Understanding the Molecular Mechanism of AD

**Figure 7:**
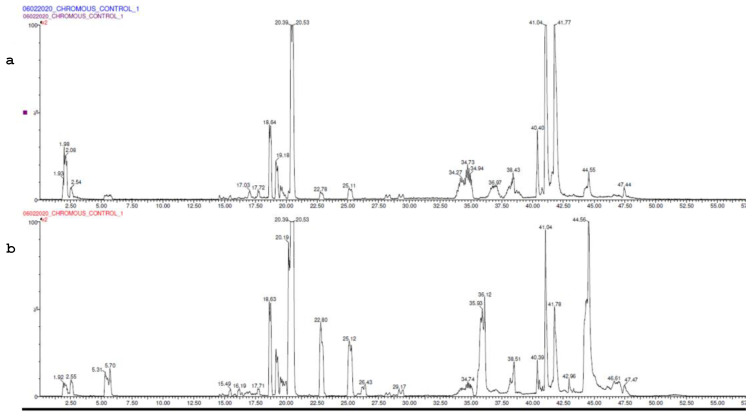
(**a**) UPLC chromatogram of control. (**b**) UPLC chromatogram of model.

LC-MS analysis was performed on the serum sample of the control group and the 60th-day sample (Figure 7a,b).

Statistical analysis was performed to retrieve the list of upregulated and downregulated proteins compared to the control. The top 20 upregulated and downregulated proteins are described in Table 2 and Table 4, respectively.

**Figure 8:**
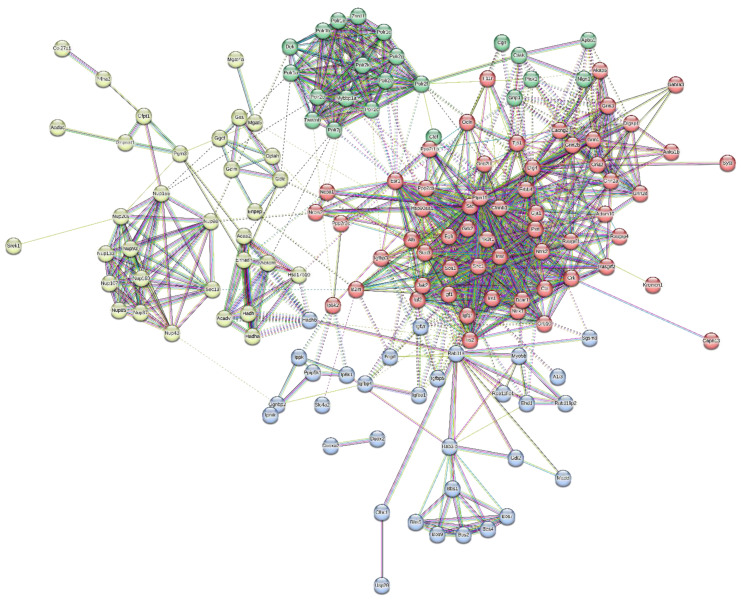
Protein interaction network of upregulated proteins dataset and its division into 4 clusters.

Further, a total of 67 proteins were observed to have been upregulated. A protein network was constructed between them, and the proteins were clustered in four clusters based on their involvement in common signalling pathways (Figure 8). Some of the major protein classes were chromatin-binding proteins, transcription factors, structural proteins, protein-modifying enzymes, etc. (Table 3).

Proteins upregulated in the serum were mostly involved in the Alzheimer’s Disease amyloid secretase pathway, androgen/oestrogen/progesterone biosynthesis, apoptosis signalling pathway, insulin/IGF pathway-protein kinase B signalling cascade, Metabotropic glutamate receptor group I pathway, Wnt signalling pathway, etc.

**Figure 9:**
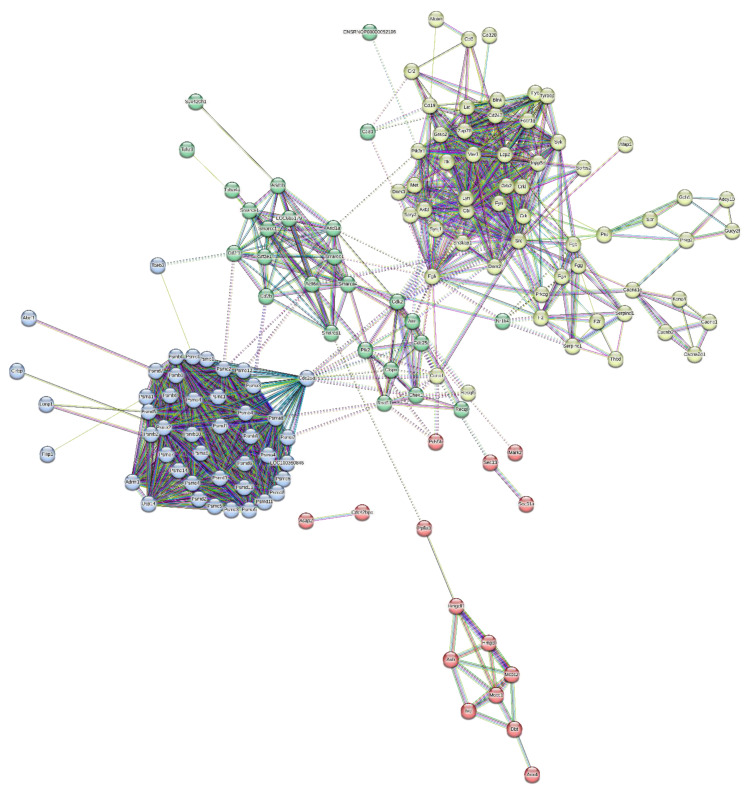
Protein interaction network of downregulated-protein dataset and its division into 4 clusters.

Similarly, a total of 64 proteins were observed to be downregulated (Table 4). The protein network was constructed between them, and the proteins were clustered in four clusters based on their involvement in common signalling pathways (Figure 9).

Some of the major protein classes included cell adhesion molecules, chaperones, chromatin-binding proteins, cytoskeletal proteins, protein-modifying enzymes, transporter proteins, etc. (Table 5).

The proteins downregulated in the serum were mostly involved the in 5HT2-type receptor-mediated signalling pathway, alpha adrenergic receptor signalling pathway, Alzheimer disease amyloid secretase pathway, Angiogenesis, B-cell activation, blood coagulation, Dopamine receptor-mediated signalling pathway, hypoxia response via HIF activation, inflammation mediated by the chemokine and cytokine signalling pathways, etc.

#### 4.2.12. Immunohistochemistry of Sporadic AD Brain and Liver Tissue

IHC was performed to validate the presence of identified proteins in both brain and liver tissue. The 60th-day brain and liver sections were chosen as they signified the severity of AD.

#### 4.2.13. Amyloid Precursor Plaques (APP)

**Figure 10:**
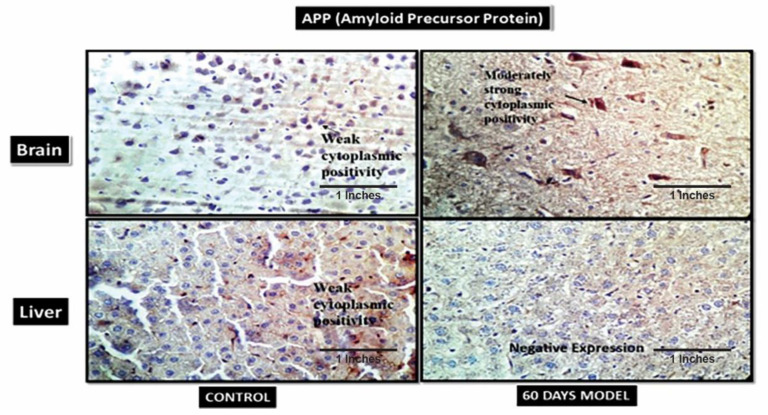
Photomicrographs of IHC images of hypothalamus section of brain and liver tissue sections of Wistar rats showing control and diseased sections stained with APP antibody (400×).

The IHC showed weak cytoplasmic APP in the control brain (Figure 10), while the sporadic AD (60th-day) brain had moderately strong cytoplasmic positivity. A few amyloid plaques can be seen in the background. Likewise, in liver sections, the control had low cytoplasmic positivity, but the 60th-day sporadic AD liver had negative APP expression. The liver is the site of amyloid clearance in body and, therefore, since the liver was dysfunctional, it was observed that it was unable to clear APP from the body, and, therefore, had negative expression in the AD model.

#### 4.2.14. Hypoxia-Inducible Factor 3-Alpha (HIF3A)

**Figure 11:**
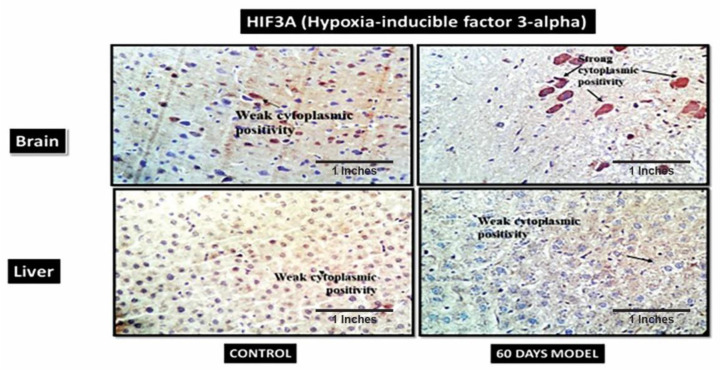
Photomicrographs of IHC images of hypothalamus section of brain and liver tissue sections of Wistar rats, showing control and diseased sections stained with HIF3A antibody (400×).

HIF3A works during hypoxic conditions. The IHC showed weak cytoplasmic positivity for HIF3A in the control brain section, while the diseased brain showed very strong cytoplasmic positivity (Figure 11). However, in the liver section, both the control and diseased states showed weak cytoplasmic activity.

#### 4.2.15. B-Cell Lymphoma 2 (BCL2)

**Figure 12:**
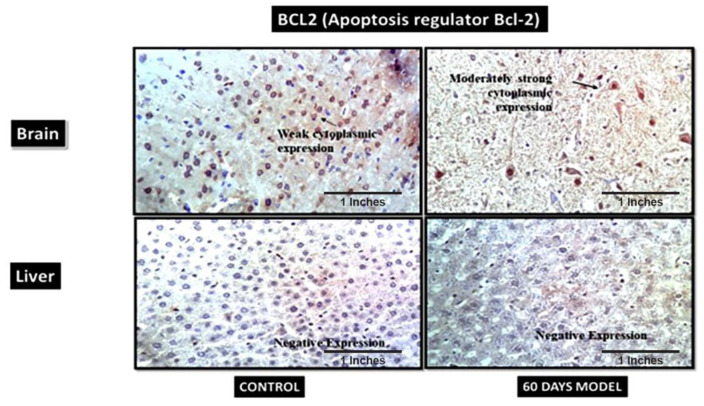
Photomicrographs of IHC images of hypothalamus section of brain and liver tissue sections of wistar rats showing control and diseased sections stained with BCL2 antibody (400×).

BCL2 is also known as an apoptosis regulator and its primary function is to prevent apoptosis. In the current model, the control brain section had weak cytoplasmic positivity for BCl2 (Figure 12), while the diseased model had moderately strong cytoplasmic expression, which might have been due to its increased role in the brain, as neurons are severely damaged and die in last stages of AD. I.e., in the later stages of LOAD, the overexpression of this gene is correlative of the tissue’s demand to stop apoptosis and save neurons from dying However, the liver sections from both the control and the AD model showed negative expression.

#### 4.2.16. Insulin Receptors (INSR)

**Figure 13:**
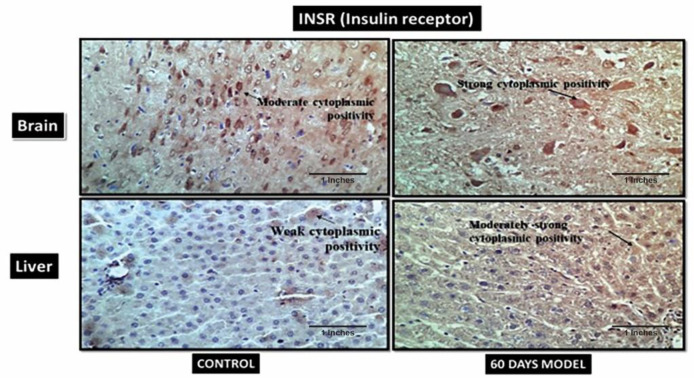
Photomicrographs of IHC images of hypothalamus section of brain and liver tissue sections of Wistar rats showing control and diseased sections stained with INSR antibody (400×).

In the current model the control brain section has moderate cytoplasmic positivity while the diseased model has strong cytoplasmic positivity (Figure 13). Similarly, the control liver section has weak cytoplasmic positivity while diseased liver has strong cytoplasmic positivity. An increase in insulin receptors is also indicative of increased glucose levels in respective tissue.

### 4.3. Discussion

Despite extensive Alzheimer’s research worldwide, still no effective medication exists for an AD cure, and the reason for this is the complexity of AD and the lack of understanding of the molecular signalling and pathways involved [18,19,20,21,22]. Recent studies have shed some light on the involvement of the liver in neurological diseases [23,24,25,26,27,28]. The link between liver diseases like primary biliary cirrhosis (PBC), non-alcoholic fatty liver disease (NAFLD), cirrhosis, Hepatocellular carcinoma (HCC) and dementia have been established [29,30,31], but the molecular link and shared pathways between the liver and brain are still unknown. Therefore, liver-based therapies in AD is a completely new domain to be explored. A few clinical studies have recently confirmed the signature of a dysfunctional liver profile in AD patients, and have showed that these symptoms appear very early and stay for a long duration of time before the actual diagnosis of AD [4,32,33]. Therefore, if the liver can be supported or treated, it can also help in ameliorating the ill effects of liver-induced cognitive impairment, as it is thought to be the one of probable reasons involved in the pathogenesis of LOAD. The most plausible reason for a long-term dysfunctional liver profile of such a huge population is also accredited to substantial junk food eating and a poor lifestyle.

The present study used Wistar rats for modelling LOAD. The model rats displayed signs of reduced cognition, as is the case with AD patients. Starting with the liver profile, initially this model displayed reduced ALT levels, which is consistent with human AD symptoms, as it is known that ALT levels decrease in human AD patients as well, and reduced ALT levels are linked to neurodegeneration [34]. The levels of AST and ALP were increased, similar to data from human AD patients. The other symptoms seen in model were bowel incontinence, urinary incontinence, a loss of muscle strength, excessive sugar in the brain tissue, etc., along with memory decline. These all are also parallel to human AD symptoms [18,19,20,21,22,23,24,25,26,27,34,35,36,37]. This model also exhibited insulin resistance. Similarly, AD patients are known to have persistent insulin resistance [38]. Glucose levels were also checked in brain and liver tissue. Glucose levels were significantly increased in the brain tissue around first month and reduced slightly in second month, but were still significantly higher compared to the control subjects. This is also in accordance with the latest data from clinical studies [35,36,37,38]. The altered expression of insulin receptors is a characteristic of LOAD [39,40]. An increase in glucose in the brain is very detrimental for the brain as it leads to increased advanced glycation end-products (AGEs) [41]. AGEs, along with amyloid beta, damages neighbouring neurons [42]. The glucose levels in liver tissue were observed to have increased in 2nd month of the LOAD model compared to the control. Further, the ALT levels in both the serum and tissue of treated rats decreased sharply in the first month and then showed a significant rise in the 2nd month. A molecular explanation for this phenomenon is not available, and this observation in the present study is a new finding. A few studies have shown that extremely low levels of AST are linked to frailty and an increased risk of mortality in the aged population [43]. Frailty here denotes a loss of skeletal muscle strength and mass [44]. AST levels were observed to gradually and significantly increase with disease progression. Generally, the serum AST/ALT ratio is used to review the condition of liver disease, and, therefore, was measured in the current model as 1.06. This shows that the current model rats of LOAD had signatures of cirrhosis, as also observed by some other studies [45]. The ALP levels increased gradually and significantly by the end of the 2nd month. The ALP levels in tissue were unusual, as they showed a significant increase at 1 month and then a significant decrease in the 2nd month. The MDA levels in the treated model were greatly increased in the 2nd month. This is indicative of excessive inflammation in the body.

Histopathological and image analyses were performed on both the liver and the hypothalamus section of the brain. The brain section retrieved from the 2nd-month LOAD model showed deformed glial nodules and apoptotic neurons. It also showed occasional degenerated neurons and a few amyloid plaques. Amyloid plaques are hallmarks of AD. Severe vacuolization in this stage within the cell matrix is also an indication of cell death. Likewise, the 2nd-month liver section showed a mildly effaced lobular architecture, an oval nucleus with coarse chromatin and prominent nucleoli, and the extravasation of RBCs. The liver showed scars and signatures of a cirrhotic liver. Image analysis confirmed the decrease in cell size of both the neuronal cells and hepatocytes, which is an indication of cell death due to LOAD.

Some of the novel observations in this model were damaged intestinal villi by the end of the 2nd month and excessive bowel and urinary incontinence in the end stages of the LOAD model, which is also in accordance to earlier reports that stated that damaged villi are indicative of less absorption of nutrients and minerals from food, which this results in malnutrition [46]. LOAD patients are also recognized for their malnutrition and disturbed gut microbiome [47]. A large part of this disturbed microbiome is dedicated to unhealthy eating and drinking [48,49]. We made a similar observation in our model regarding disease progression, wherein excessive bowl incontinence lead to the death of a subject.

Nano-LCMS was performed on a serum sample from a 2nd-month subject. Statistical analysis revealed that 67 proteins were upregulated and involved in biological pathways like the Alzheimer’s Disease amyloid secretase pathway, androgen/oestrogen/progesterone biosynthesis, apoptosis signalling pathway, insulin/IGF pathway-protein kinase B signalling cascade, Metabotropic glutamate receptor group I pathway, Wnt signalling pathway, etc. Similarly, a total of 64 proteins were downregulated and were involved in biological processes, like the 5HT2-type receptor-mediated signalling pathway, alpha adrenergic receptor signalling pathway, Alzheimer disease amyloid secretase pathway, Angiogenesis, B-cell activation, blood coagulation, the Dopamine receptor-mediated signalling pathway, hypoxia response via HIF activation, inflammation mediated by the chemokine and cytokine signalling pathways, etc. These findings are not present in the literature, and this study reports it for the first time.

Additionally, immunohistochemistry showed a moderately strong presence of APP in the diseased brain compared to the control. This hallmark confirms an AD pathology. Also, the control liver showed the presence of amyloid plaques in the tissue, but it was observed that diseased liver had a negative expression, contrary to the control liver. This is suggestive of the fact that the liver is also the site of peripheral Aβ clearance from systemic circulation [50]. When the liver gets damaged, as in the case of LOAD, it stops clearing amyloid plaques from the body; hence, this further results in an increased load of Aβ plaques in systemic circulation, as well as in the brain. Therefore, the hypothesis generated for the liver–brain axis in LOAD was, hence, validated by this model. The IHC results of hypoxia-Inducible Factor 3-Alpha (HIF3A) antibody showed weak positivity in the control samples but very strong positivity in the treated sample. HIF3A works under the circumstances of hypoxia. Its positivity in the treated sample demonstrates hypoxic conditions in the AD brain. A recent study also confirmed the role of HIF3A-induced hypoxia in human and animal AD model studies [51]. The control and treatment livers both showed weak cytoplasmic positivities. The probable explanation for weak cytoplasmic positivity in the treated liver could be due to the damage in the liver, which is then unable to combat the hypoxic stress. B-cell lymphoma 2 (BCL2) is an apoptosis regulator and prevents apoptosis. The control brain section had weak cytoplasmic positivity for BCl2, while the diseased model had a moderately strong cytoplasmic expression, which might be due to its increased role in the brain, as neurons are severely damaged and die in the last stages of AD. A similar finding was also reported by a clinical study [52,53]. However, the liver sections treated in the AD liver model showed negative expression. A few clinical studies have also reported that in hepatitis, BCL2 expression is absent [54]. The IHC results of insulin receptors (INSR) showed moderate cytoplasmic positivity in the control but a strong cytoplasmic positivity in the diseased brain. Increased INSR levels denote an increased influx of sugar into the brain. A few clinical studies have also shown an increased amount of glucose in AD patients’ brains [35,36]. There was an increased expression of INSR in liver tissue as well, which also denotes the upregulation of glucose in the liver tissue. The mice in our model displayed significant elevations of glucose in liver tissue as well. These data are novel and have not been reported anywhere so far.

## 5. Conclusions

This study highlights the intricate relationship between Alzheimer’s Disease (AD) and liver health, offering crucial insights into this complex neurological condition. Despite extensive research worldwide, AD remains without effective curative treatments due to its multifaceted nature and poorly understood molecular pathways. Recent research has illuminated a compelling link between liver conditions, such as primary biliary cirrhosis (PBC), non-alcoholic fatty liver disease (NAFLD), cirrhosis, Hepatocellular Carcinoma (HCC), and dementia, especially in the context of AD. Clinical studies have revealed early signs of liver dysfunction in AD patients, potentially enabling early diagnosis and intervention. Unhealthy lifestyle factors, notably poor dietary choices, seem to exacerbate long-term liver issues in AD.

Using a Wistar-rat model to mimic Late-Onset Alzheimer’s Disease (LOAD), this study replicated key AD features, including altered liver enzyme levels, muscle weakness, and elevated brain and liver glucose levels, aligning with the observed insulin resistance in AD. Notably, heightened brain glucose levels contribute to advanced glycation end-product (AGE) formation and potential neuronal damage. Altered insulin-receptor expression, characteristic of LOAD, mirrors elevated brain glucose levels. Histopathological and image analyses validated the model’s accuracy, confirming amyloid plaques in the brain and signs of cirrhosis in the liver, suggesting cellular changes and cell death linked to LOAD progression. Unique findings, such as damaged intestinal villi, bowel and urinary incontinence, and malnutrition in advanced LOAD stages, parallel issues seen in AD patients, emphasizing the role of unhealthy eating habits. Additionally, Nano-LCMS analysis revealed novel proteins and biological pathways associated with LOAD, providing fresh insights. Immunohistochemistry suggests the liver’s role in clearing amyloid plaques and impacting AD pathogenesis.

In conclusion, this study represents a significant step in understanding the intricate connection between liver and brain health in LOAD. While questions persist, these findings open promising avenues for research and potential therapies targeting the liver-related aspects of AD. Unveiling the molecular mechanisms behind these observations holds promise for future advances in AD treatment and management.

## Figures and Tables

**Table 1:** Analysis of histopathological images of Wistar-rat brain and liver treated by DGalN + Ethanol + D-Galact using Image J software 2.9.0 1.52P.

Sample	Total Area of Cell	Average Size of Cell
Hypothalamus region of control subject	2,716,249	666.237
Hypothalamus region of 30th-day DGalN + Ethanol + D-Galact-treated brain sample	3,297,864	207.074
Hypothalamus region of 60th-day DGalN + Ethanol + D-Galact-treated brain sample	380,193	93.551
Liver section of Control subject	474,868	669.772
Liver section of 30th-day DGalN + Ethanol + D-Galact-treated liver sample	457,997	151.454
Liver section of 60th-day DGalN + Ethanol + D-Galact-treated liver sample	252,249	47.088

**Table 2:** Top 20 upregulated proteins retrieved after Nano LC-MS of control and 60-day sample comparison.

Accession	Description	Score	Treated_R1:Control_1_Ratio
Q5GH61	Membrane transport protein XK OS = Rattus norvegicus OX = 10,116 GN = Xk PE = 1 SV = 1	350.73	2.117
Q9QZH8	Arylacetamide deacetylase OS = Rattus norvegicus OX = 10,116 GN = Aadac PE = 2 SV = 3	271.41	2.203396
A2RUV9	Adipocyte enhancer-binding protein 1 OS = Rattus norvegicus OX = 10,116 GN = Aebp1 PE = 2 SV = 1	249.25	2.691234
P48768	Sodium/calcium exchanger 2 OS = Rattus norvegicus OX = 10,116 GN = Slc8a2 PE = 1 SV = 1	247.74	2.054433
O35430	Amyloid-beta A4 precursor protein-binding family A member 1 OS = Rattus norvegicus OX = 10,116 GN = Apba1 PE = 1 SV = 1	207.95	2.316367
P45953	Very long-chain-specific acyl-CoA dehydrogenase, mitochondrial OS = Rattus norvegicus OX = 10,116 GN = Acadvl PE = 1 SV = 1	188.83	3.743422
Q6AXS3	Protein DEK OS = Rattus norvegicus OX = 10,116 GN = Dek PE = 1 SV = 1	177.55	2.801066
Q62645	Glutamate receptor ionotropic, NMDA 2D OS = Rattus norvegicus OX = 10,116 GN = Grin2d PE = 1 SV = 2	177.43	2.033991
Q62878	3 beta-hydroxysteroid dehydrogenase/Delta 5-- > 4-isomerase type 4 OS = Rattus norvegicus OX = 10,116 GN = Hsd3b6 PE = 2 SV = 4	173.16	2.117
O08873	MAP kinase-activating death domain protein OS = Rattus norvegicus OX = 10,116 GN = Madd PE = 1 SV = 1	160.7	4.13712
P50399	Rab GDP dissociation inhibitor beta OS = Rattus norvegicus OX = 10,116 GN = Gdi2 PE = 1 SV = 2	148.93	4.0552
Q08834	Alpha-1,6-mannosylglycoprotein 6-beta-N-acetylglucosaminyltransferase A OS = Rattus norvegicus OX = 10,116 GN = Mgat5 PE = 1 SV = 1	147.12	2.159766
Q9R1D1	Transcriptional repressor CTCF OS = Rattus norvegicus OX = 10,116 GN = Ctcf PE = 2 SV = 1	140.48	2.117
A2RRT9	Cytochrome P450 4V2 OS = Rattus norvegicus OX = 10,116 GN = Cyp4v2 PE = 2 SV = 1	139.86	2.339647
Q6AYM0	Uncharacterized protein C7orf31 homolog OS = Rattus norvegicus OX = 10,116 PE = 1 SV = 1	137.69	2.4109
P20236	Gamma-aminobutyric acid receptor subunit alpha-3 OS = Rattus norvegicus OX = 10,116 GN = Gabra3 PE = 1 SV = 1	133.29	8.499439
Q3B7T9	Rab11 family-interacting protein 1 OS = Rattus norvegicus OX = 10,116 GN = Rab11fip1 PE = 1 SV = 1	129.98	2.138276
A0A0G2K2P5	Tight junction protein ZO-1 OS = Rattus norvegicus OX = 10,116 GN = Tjp1 PE = 1 SV = 1	129.89	2.316367
D3ZJ96	Ubiquitin carboxyl-terminal hydrolase 28 OS = Rattus norvegicus OX = 10,116 GN = Usp28 PE = 2 SV = 2	119.99	2.459603
Q5XIR8	Clathrin heavy chain linker domain-containing protein 1 OS = Rattus norvegicus OX = 10,116 GN = Clhc1 PE = 2 SV = 1	111.48	2.159766

**Table 3:** K-means clustering on upregulated protein networks (only 30 displayed here).

Clustering Method	Cluster Number	Cluster Color	Protein Name
kmeans	4	Blue	Hsd3b6
kmeans	2	Yellow	Hsd17b10
kmeans	2	Yellow	Oplah
kmeans	4	Blue	Adamts1
kmeans	2	Yellow	Acaa2
kmeans	4	Blue	Afap1l1
kmeans	2	Yellow	Acadm
kmeans	2	Yellow	Acadvl
kmeans	2	Yellow	Aebp1
kmeans	1	Red	Akap5
kmeans	2	Yellow	Mgat5
kmeans	4	Blue	A1i3
kmeans	3	Green	Apba1
kmeans	1	Red	Anks1b
kmeans	2	Yellow	Aadac
kmeans	4	Blue	Bbs1
kmeans	4	Blue	Bbs2
kmeans	4	Blue	Bbs4
kmeans	4	Blue	Bbs5
kmeans	4	Blue	Bbs7
kmeans	4	Blue	Bbs9
kmeans	1	Red	Ntrk2
kmeans	1	Red	B2m
kmeans	1	Red	Bcar1
kmeans	1	Red	Cacng2
kmeans	3	Green	Cask
kmeans	1	Red	Capn13
kmeans	1	Red	Ctnnb1
kmeans	3	Green	Cgn
kmeans	4	Blue	Clhc1

**Table 4:** Top 20 downregulated proteins retrieved after Nano-LC-MS of control and 60th-day sample comparison.

Accession	Description	Score	Treated_R1:Control_1_Ratio
Q64595	cGMP-dependent protein kinase 2 OS = Rattus norvegicus OX = 10,116 GN = Prkg2 PE = 1 SV = 1	40.44	0.029896915
Q9Z286	Adenylate cyclase type 10 OS = Rattus norvegicus OX = 10,116 GN = Adcy10 PE = 1 SV = 1	70.17	0.035436961
P97573	Phosphatidylinositol 3,4,5-trisphosphate 5-phosphatase 1 OS = Rattus norvegicus OX = 10,116 GN = Inpp5d PE = 1 SV = 1	66.43	0.095369171
Q5XIT9	Methylcrotonoyl-CoA carboxylase beta chain, mitochondrial OS = Rattus norvegicus OX = 10,116 GN = Mccc2 PE = 2 SV = 1	69.03	0.099261257
A1A5Q6	Sperm motility kinase OS = Rattus norvegicus OX = 10,116 GN = Smok PE = 2 SV = 1	31.15	0.099261257
Q5FVC7	Arf-GAP with coiled-coil, ANK repeat and PH domain-containing protein 2 OS = Rattus norvegicus OX = 10,116 GN = Acap2 PE = 1 SV = 1	201.35	0.140858416
Q562B4	Nucleus accumbens-associated protein 2 OS = Rattus norvegicus OX = 10,116 GN = Nacc2 PE = 2 SV = 2	52.05	0.142274065
O35413	Sorbin and SH3 domain-containing protein 2 OS = Rattus norvegicus OX = 10,116 GN = Sorbs2 PE = 1 SV = 2	37.21	0.146606968
Q63148	Chordin OS = Rattus norvegicus OX = 10,116 GN = Chrd PE = 2 SV = 2	29.63	0.151071811
B4F7E8	Niban-like protein 1 OS = Rattus norvegicus OX = 10,116 GN = Fam129b PE = 1 SV = 1	105.4	0.152590106
Q8VH46	Actin filament-associated protein 1 OS = Rattus norvegicus OX = 10,116 GN = Afap1 PE = 1 SV = 1	69.74	0.165298896
P62634	Cellular nucleic acid-binding protein OS = Rattus norvegicus OX = 10,116 GN = Cnbp PE = 2 SV = 1	189.19	0.195929575
D4ACP5	ATP-dependent DNA helicase Q5 OS = Rattus norvegicus OX = 10,116 GN = Recql5 PE = 1 SV = 1	54.58	0.216535674
Q9R012	Serine/threonine-protein kinase PLK2 OS = Rattus norvegicus OX = 10,116 GN = Plk2 PE = 1 SV = 1	56.66	0.218711891
Q924S5	Lon protease homolog, mitochondrial OS = Rattus norvegicus OX = 10,116 GN = Lonp1 PE = 2 SV = 1	73.71	0.22313016
P85107	Trimethylguanosine synthase OS = Rattus norvegicus OX = 10,116 GN = Tgs1 PE = 1 SV = 1	113.15	0.225372653
Q925Q9	SH3 domain-containing kinase-binding protein 1 OS = Rattus norvegicus OX = 10,116 GN = Sh3kbp1 PE = 1 SV = 2	165.95	0.227637684
P17220	Proteasome subunit alpha type-2 OS = Rattus norvegicus OX = 10,116 GN = Psma2 PE = 1 SV = 3	291.98	0.239308935
Q6TRW4	Sister chromatid cohesion protein PDS5 homolog B OS = Rattus norvegicus OX = 10,116 GN = Pds5b PE = 1 SV = 2	58.57	0.251578554
Q924S1	1-acyl-sn-glycerol-3-phosphate acyltransferase delta OS = Rattus norvegicus OX = 10,116 GN = Agpat4 PE = 2 SV = 1	104.5	0.254106958

**Table 5:** K-means clustering on downregulated protein networks (only 30 displayed here).

Clustering Method	Cluster Number	Cluster Color	Protein Name
kmeans	4	Blue	Psma7
kmeans	4	Blue	Psma8
kmeans	4	Blue	Psma5
kmeans	4	Blue	Psmb3
kmeans	4	Blue	Psmb5
kmeans	4	Blue	Psmb1
kmeans	4	Blue	Psmb4
kmeans	4	Blue	Psmb9
kmeans	4	Blue	Psmd13
kmeans	4	Blue	Psmd2
kmeans	4	Blue	Psmc1
kmeans	4	Blue	Psmc3
kmeans	4	Blue	Psmc4
kmeans	4	Blue	Psmc5
kmeans	4	Blue	Psmd8
kmeans	4	Blue	Psmd1
kmeans	4	Blue	Psmc2
kmeans	4	Blue	Psmc6
kmeans	1	Red	Hmgcll1
kmeans	1	Red	Mccc1
kmeans	2	Yellow	Pts
kmeans	2	Yellow	Afap1
kmeans	3	Green	Actl6a
kmeans	2	Yellow	Adcy10
kmeans	1	Red	Aox4
kmeans	1	Red	Acap2
kmeans	3	Green	Atm
kmeans	4	Blue	Abcf1
kmeans	3	Green	Recql
kmeans	2	Yellow	Recql5

## Data Availability

Data is contained within the article.
